# Symptom-based screening tool for asthma syndrome among young children in Uganda

**DOI:** 10.1038/s41533-020-0175-1

**Published:** 2020-05-06

**Authors:** Rebecca Nantanda, Volkert Siersma, Grace Ndeezi, James K. Tumwine, Marianne S. Østergaard

**Affiliations:** 1 Makerere University Lung Institute, Makerere University College of Health Sciences, Kampala, Uganda; 2 The Research Unit for General Practice and Section of General Practice, Department of Public Health, University of Copenhagen, Copenhagen, Denmark; 3 Department of Paediatrics and Child Health, Makerere University College of Health Sciences, Kampala, Uganda

**Keywords:** Asthma, Asthma

## Abstract

Under-diagnosis of asthma in ‘under-fives’ may be alleviated by improved inquiry into disease history. We assessed a questionnaire-based screening tool for asthma among 614 ‘under-fives’ with severe respiratory illness in Uganda. The questionnaire responses were compared to post hoc consensus diagnoses by three pediatricians who were guided by study definitions that were based on medical history, physical examination findings, laboratory and radiological tests, and response to bronchodilators. Children with asthma or bronchiolitis were categorized as “asthma syndrome”. Using this approach, 253 (41.2%) had asthma syndrome. History of and present breathing difficulties and present cough and wheezing was the best performing combination of four questionnaire items [sensitivity 80.8% (95% CI 77.6–84.0); specificity 84.7% (95% CI 81.8–87.6)]. The screening tool for asthma syndrome in ‘under-fives’ may provide a simple, cheap and quick method of identifying children with possible asthma. The validity and reliability of this tool in primary care settings should be tested.

## Introduction

Childhood asthma is common but is largely under-diagnosed, while bacterial pneumonia is over-diagnosed^1–4^. Among children under five years of age (‘under-fives’ or U-5s) the signs and symptoms of acute asthma and pneumonia overlap, and this creates difficulty in distinguishing asthma from pneumonia even among the experienced^2,3,5^. Consequently, many children with asthma are misdiagnosed as pneumonia. Over-diagnosis of pneumonia is associated with over-use of antibiotics and worsening antibiotic resistance and withholding asthma medicines for children who need them^6–9^. Failure to diagnose and treat asthma is associated with repeated hospital visits, avoidable high healthcare costs and low quality of life of affected children and their families^10,11^ Un-treated asthma also affects growth and development of the affected children^12,13^. Furthermore, several studies have reported that the pathologic features of asthma; inflammation and basement membrane thickening, start before 3 years of age^14^. Hence, early identification of asthma may prevent permanent airway changes^15^.

Epidemiologically, children with wheeze have been retrospectively categorized into three; (1) transient, (2) persistent and (3) late onset based on cohort data. However, this classification has no clinical significance because it does not provide guidance on how and when to intervene^16,17^. Recently, two wheezing phenotypes among young children have been described according to temporal symptom patterns, and these are; (1) episodic viral wheeze which refers to discrete periods of wheezing associated with symptoms of a viral cold only, and multiple-trigger wheeze in which wheezing is present with or without viral episodes^16^. Multiple-trigger wheeze responds to short-acting bronchodilators (SABA), possibly due to underlying asthma. However, identifying these children requires recognition of the wheezing episodes by parents/caretakers so that they can narrate them to clinicians during consultation. However, this is challenging because of poor recognition of wheeze by caretakers^18–21^. Therefore, using this approach alone may not comprehensive identify children with possible/at risk of asthma, hence the need for a wider scope of symptoms to assess.

Acute bronchiolitis, especially due to rhinovirus and to a lesser extent RSV is a risk factor for development of asthma later in childhood^22–25^. Different studies have indicated that up to 30% of children hospitalized with acute bronchiolitis develop asthma, and that such children may respond to SABA, possibly due to underlying hyper-responsive airways^26–28^. A pragmatic approach that highlights this possibility is therefore useful in increasing the index of suspicion of asthma in children, such that they can be diagnoseds and managed early.

The diagnosis of asthma, and indeed other airway diseases such as pneumonia, in young children is very dependent on inquiry into the clinical history of the disease process. There is a lack of useable diagnostic tests for airflow limitation and airway inflammation in this age group. Other diagnostic tools such as X-ray may not be readily available in primary care health facilities and may require extensive travel with a sick child. This is aggravated in rural areas and in low-income countries where even availability of health care specialists may be limited. For many, a clear and detailed history on the disease presentation is the most reasonable first-line approach to determining diagnosis and treatment.

Using the Integrated Management of Childhood Illnesses (IMCI) guidelines^29^, the World Health Organization (WHO) attempts to use the symptom of wheeze to diagnose asthma in young children but this does not perform well and many children with asthma remain undiagnosed^1^. Furthermore, the IMCI guidelines use wheezing as the only symptom to diagnose asthma, yet, studies have reported that the symptom of wheeze is poorly understood by both caretakers and health workers^19,30^. Hence, there is a need for a more comprehensive overview of the use of information on the clinical history and presentation to identify children with probable asthma. The objective of the present paper is to assess the performance of a screening tool for asthma among children <5 years of age, based on inquiry into symptoms and signs.

## Results

### General overview

Six hundred and fourteen children (614) were included in the study. The majority (80.2%) of the children were aged 24 months and below. The median age was 10 months (Interquartile range: 6–18). Three hundred and fourty seven (347) (56.5%) were boys. Of the 614 participants, 468 (76.0%) were from urban settings. The number of children who were diagnosed with asthma or bronchiolitis were categorized as “asthma syndrome” and contributed 253 (41.2%) participants. Of the 253 children who were diagnosed with asthma syndrome, 125 (49.4%) had acute bronchiolitis while 78 (30.8%) had asthma alone and 50 (19.8%) had a combination of asthma and bacterial pneumonia. The details of the diagnoses are provided in Table 1.

**Table 1:** Demographic characteristics of the study participants (*N* = 614).

Diagnosis	Total (*n*)	Percentage (%)	Age group (months)		Sex		Residence	
			<12	≥12	M	F	Urban	Rural
Bronchiolitis	125	20.4	109	16	85	40	99	26
Asthma	78	12.7	20	58	42	36	64	14
Combined asthma and bacterial pneumonia	50	8.1	17	33	29	21	37	13
Bacterial pneumonia	167	27.2	77	90	95	72	129	38
Viral pneumonia	163	26.5	92	71	82	81	122	41
Others^a^	31	5.0	18	13	14	17	17	14
Total	614	100	333	281	347	267	468	146

^a^Pulmonary tuberculosis, pneumocystis jirovecii pneumonia.

Response to bronchodilator therapy was one of the criteria used for diagnosis of asthma, and of the 253 children with asthma syndrome, 128 (50.6%) of them responded to bronchodilators. The remaining 125 children did not respond to bronchodilators and were categorized as having bronchiolitis.

### Individual APQ questionnaire items to identify children with asthma syndrome

Table 2 presents the ten items of the 88 in the Ugandan APQ questionnaire that have the best diagnostic properties with respect to the AUC. Only the item that asks about present wheezing has properties that are reasonably acceptable. Many of the other top items also ask about wheezing in some form, but here the sensitivity is far below acceptable. We observed that even with the top item more than a sixth of the children (17.4%) were misclassified.

**Table 2:** Diagnostic properties of the top-10 (according to AUC) individual APQ items for the diagnosis of asthma syndrome in U-5s.

	Sensitivity%	Specificity%	AUC%	PPV%	NPV%	MCE%
	(95% CI)	(95% CI)	(95% CI)	(95% CI)	(95% CI)	(95% CI)
Your child’s disease today: wheezing/whistling?	80.2 (75.0–84.8)	84.2 (80.2–87.7)	82.2 (79.1–85.3)	78.1 (72.8–82.8)	85.9 (82.0–89.2)	17.4 (14.6–20.6)
What symptoms have your child had in the last few days (before coming to the hospital): wheezing/whistling?	50.6 (44.4–56.7)	91.1 (87.9–93.8)	70.9 (67.5–74.3)	80.0 (73.4–85.7)	72.5 (68.2–76.4)	25.6 (22.2–29.1)
Your child’s disease. How did it begin: wheezing/whistling?	36.8 (31.0–42.8)	94.2 (91.5–96.3)	65.5 (62.3–68.7)	81.6 (73.8–73.8)	68.0 (63.8–72.0)	29.5 (26.0–33.2)
In the last 3 months before coming to hospital, has your child: wheezing/whistling?	34.4 (28.7–40.4)	92.8 (89.9–95.2)	63.6 (60.4–66.8)	77.0 (68.7–84.1)	66.9 (62.7–70.9)	31.3 (27.7–35.0)
Earlier problems (since birth): does your child wake up because of wheezing?	29.6 (24.2–35.5)	95.6 (93.1–97.4)	62.6 (59.6–65.6)	82.4 (73.8–89.3)	66.0 (61.8–69.9)	31.6 (28.0–35.3)
Earlier problems (since birth): has your child had recurrent episodes of wheezing/whistling?	32.4 (26.8–38.3)	91.1 (87.9–93.8)	61.8 (58.5–65.0)	71.9 (63.3–79.6)	65.8 (61.6–69.9)	33.1 (29.4–36.8)
Earlier problems (since birth): does the wheezing usually occur in the night/early morning?	26.5 (21.3–32.1)	95.3 (92.8–97.2)	60.9 (58.0–63.8)	79.8 (70.3–87.4)	64.9 (60.8–68.9)	33.1 (29.4–36.8)
What symptoms has your child had in the last few days (before coming to the hospital): did your child wake up because of difficulty breathing?	60.9 (54.8–66.8)	60.7 (55.6–65.6)	60.8 (56.9–64.7)	52.0 (46.3–57.7)	68.9 (63.6–73.8)	39.3 (35.4–43.2)
Earlier problems (since birth): has your child ever taken medicine for asthma or wheezing?	27.3 (22.0–33.0)	94.2 (91.5–96.3)	60.7 (57.7–63.7)	76.7 (67.3–84.6)	64.9 (60.7–68.9)	33.4 (29.7–37.2)
Your child’s disease How did it begin: did the disease specifically start with changes in breathing?	47.4 (41.3–53.6)	73.7 (69.0–78.0)	60.6 (56.7–64.4)	55.8 (49.1–62.4)	66.7 (61.9–71.2)	37.1 (33.4–41.0)

### Sets of APQ items to screen for asthma syndrome in young children

In an attempt to improve on the performance of the single items for screening for asthma, combinations of items were investigated to assess if the diagnostic properties can be improved. Table 3 presents the combination of four items that performed best. This combination had a sensitivity of 78.3% (95% CI 75.1–81.6), a specificity of 86.6% (95% CI 83.9–89.3), AUC of 82.5% (95% CI 80.4–84.6) and a MCE of 17.0% (95% CI 15.2–19.3). This is only a marginal improvement on the single wheezing item presented in Table 2.

**Table 3:** The best performing set of four items for detection of asthma syndrome in U-5s with acute respiratory symptoms.

Disease start with difficulty in breathing?	Did painkillers help much?	Your child’s disease today: wheezing/whistling?	Did your child had significant cough in the last few days (just before coming to the hospital)?	*n*	Indicated diagnosis	Without asthma in data (%)	With asthma in data (%)
No	No	No	No	1	Asthma	0.0	100.0
Yes	No	No	No	2	No asthma	50.0	50.0
No	Yes	No	No	0			
Yes	Yes	No	No	3	No asthma	100.0	0.0
No	No	Yes	No	0			
Yes	No	Yes	No	2	Asthma	0.0	100.0
No	Yes	Yes	No	0			
Yes	Yes	Yes	No	0			
No	No	No	Yes	127	No asthma	86.6	13.4
Yes	No	No	Yes	40	No asthma	85.0	15.0
No	Yes	No	Yes	147	No asthma	87.8	12.2
Yes	Yes	No	Yes	34	No asthma	79.4	20.6
No	No	Yes	Yes	78	Asthma	17.9	82.1
Yes	No	Yes	Yes	76	Asthma	17.1	82.9
No	Yes	Yes	Yes	57	Asthma	31.6	68.4
Yes	Yes	Yes	Yes	47	Asthma	25.5	74.5

The analyses were repeated after stratifying the participants by age; 492 (80.1%) children <2 years of which 192 (39.0%) had asthma syndrome, and 122 (19.1%) aged 2–5 years of which 61 (50.0%) had asthma syndrome. For both age groups, wheezing was the best performing single item in screening for children with possible asthma with sensitivity 77.1% (95% CI 70.8–82.6) and specificity 83.7% (95% CI 79.2–87.6) for children <2 years, and sensitivity 90.2% (95% CI 81.1–96.0) and specificity 88.5% (82.9–94.1) for children aged 2 up to 5 years. Also for both age groups, as for the complete data, wheezing was part of the best performing combination of four items and the performance of the combinations was not better than the performance of the single wheeze item: sensitivity 75.4% (95% CI 71.6–79.2), specificity 85.7% (95% CI 82.6–88.7) for children <2 years and sensitivity 93.3% (95% CI 88.9–97.8), specificity 91.9% (95% CI 87.1–96.8) for children 2–5 years. The sensitivity and specificity of both the individual wheeze item and the best performing combination was higher for children aged 2 up to 5 years.

## Discussion

In this study, we aimed at investigating the use of inquiry into symptoms and signs for the screening for asthma syndrome in U-5s when presenting with cough and/or difficult breathing. The questionnaire comprised items regarding acute and chronic respiratory symptoms.

Generally, the findings indicate that it is possible to correctly classify children with probable asthma using only the clinical history in up to 80% of cases. In low-income settings where there are limited skilled personnel and diagnostics, and under-diagnosis of asthma^3,29,31^, these symptoms can be very useful in guiding the process of screening and subsequent diagnosis of asthma among children <5 years. Although wheeze is one of the major symptoms of asthma in children, it is poorly understood and expressed by caretakers in many settings^18-20^. This could explain why the symptom of wheeze alone did not perform any better than the combination of symptoms.

The results show that using a set of items such as cough, wheezing and breathing difficulties, and importantly, previous attacks of breathing difficulties, we can correctly identify more than three-quarters (81%) of children with asthma syndrome. Some studies have attempted to develop tools for screening and/or diagnosis of pediatric asthma. However, these studies were done in older children and in high income countries^32,33^. There is scarcity of data on tools for detection of asthma in young children, yet, they are the most affected by asthma symptoms^4,34,35^. A few studies have identified symptoms that can be used for the diagnosis of asthma in children <5 years^5,36^. In India, Sachdev and others studied children aged 2–59 months and identified two symptoms that were predictive of asthma; (1) previous episodes of cough and difficulty in breathing and (2) having had no fever at the time of presentation with acute respiratory symptoms^5^. Another study by Gowraiah et al.^36^ reported that a history of previous episodes of acute respiratory symptoms and wheeze were predictive of wheezy disease among young children, and that such children were more likely to have asthma rather than pneumonia. The findings in this study are similar to those of previous studies and emphasize the need to look at a combination of acute and chronic/recurrent asthma symptoms in asthma case detection.

The symptoms that were identified are also closely related to the definition of asthma in young children as outlined in various international guidelines such as GINA^37^. This points to the fact that they can be applicable in clinical practice, and should be considered for validation and subsequent adaptation into primary care guidelines such as IMCI^29^. Furthermore, these tools are based on symptoms, and this is in conformity with the syndromic/symptom-based approach in screening and diagnosis of childhood illnesses that has been found to be feasible and useful in settings such as Uganda, which have limited human resources for health and diagnostics^38^. Such tools will go a long way in helping healthcare workers in low-income settings to identify and refer children with probable asthma syndrome for further evaluation.

Most individual symptoms had very low sensitivity or specificity, indicating the limitations associated with one symptom to screen for asthma in children. However, the symptom of wheeze performed well in predicting asthma syndrome. The performance did not improve when combinations of symptoms were considered. Further analysis after stratifying the children into those <2 years and 2 years up to 5 years also showed that the symptom of wheeze was important in screening children for asthma syndrome in both age groups. This indicates that for children <5 years, the symptom of wheeze is key in identifying children who may have asthma. This is similar to the approach used in the IMCI guidelines where it is used to identify children with asthma. However, understanding and expressing the symptom of wheeze by caretakers remains a challenge^18–21^ indicating the need for further research into simple items to guide the diagnosis of asthma syndrome in U-5s.

The methodological approach to this study provided for a comprehensive process of developing the questionnaire items, piloting them for understanding and relevance and using a diagnostic criteria for asthma and pneumonia based on international guidelines and incorporating laboratory and radiological test results. The APQ was developed using an iterative process including pre-testing in Uganda. This comprehensive process ensured that the scope of questionnaire items was wide and relevant to the study setting. However, some of the asthma questionnaire items, particularly those referring to recurrence of symptoms were prone to recall bias. The diagnostic process was based on clinical assessment supported by laboratory and radiological tests, and response to treatment, which is in line with international guidelines for diagnosis of asthma in children^37^. Therefore, the diagnoses made were deemed accurate.

The study had a large sample size, was rigorous and used a pragmatic approach to defining asthma syndrome which is relevant to many low-income settings and therefore the results would be useful in similar settings. However, they can only be generalized to tertiary care settings and are not directly applicable to rural and other primary care settings, because the study site was a tertiary care hospital where patients are very heterogeneous with many children (75%) from urban settings. In addition, the acute symptoms such as wheezing may be performing well because of the hospital setting where doctors may be explicitly using the term wheezing, which may not be the case in primary care settings. Therefore, the wheezing item may be performing well artificially. Lastly, the IMCI guidelines were developed for primary care settings with limited diagnostic capabilities, therefore, the tools identified in this study need to be validated in primary care settings before adaptation.

The results from this study show that the sensitivity and specificity of the screening tool for children with asthma syndrome were acceptable: the items of previous attacks of breathing difficulties and presently cough, wheezing and breathing difficulties can correctly identify more than three-quarters (81%) of under-fives with asthma syndrome. It is envisaged that this tool can provide a simple, cheap and quick method of identifying children with possible asthma syndrome. A study to test the validity and reliability of this tool is recommended to test its usefulness in primary care settings where there are limited skilled personnel and diagnostics. Screening for asthma using this approach would be very helpful in clinical practice and contribute towards mitigating the problem of under-diagnosis of asthma.

## Methods

### Study design, setting and recruitment

This was a cross-sectional survey among U-5s presenting with respiratory symptoms at the pediatric emergency unit of Mulago Hospital in Kampala. Mulago hospital is the Ugandan national referral hospital and the teaching hospital of Makerere University College of Health Sciences. At the time of the study, the daily attendance at the pediatric emergency unit was 50–70 children, and 25% of these presented with cough and/or difficult breathing. The hospital was selected as the study site because of its ability to handle laboratory and radiological investigations for diagnosis of asthma and pneumonia, facilities that are not readily available in other public hospitals in the country.

The process of recruiting participants into the study has been described in detail elsewhere^39^. Briefly, between August 2011 and July 2012, children aged 2–59 months who presented with cough and/or difficult breathing, and fast breathing were eligible for inclusion. Children with heart conditions or cardiac failure secondary to severe anemia, based on the caretaker’s history, physical examination findings and medical records, were excluded. Following informed written consent from the caretaker, the Asthma-Pneumonia Questionnaire (APQ) was administered followed by a physical examination. Children who had wheezing received nebulized salbutamol. They had their response recorded, and systemic steroids were given to those who had moderate or severe symptoms. Children who were suspected to have pneumonia were given antibiotics according to the hospital protocol^40^.

### The APQ questionnaire

The Asthma-Pneumonia Questionnaire (APQ) was developed in a qualitative study in Denmark through an iterative process with clinical experts^13^. At the outset, six dimensions of interest for U-5s with cough or breathing difficulty were identified: complaints at presentation, history of symptoms, history of asthma medicine use, family history of asthma and environmental factors. The final version of this Danish questionnaire contained 60 items focusing on the medical history, to be filled out by the child’s primary caretaker. The development of the Danish APQ questionnaire was done in Denmark spearheaded by MSO. It was then adapted for use in Uganda by RN. The process of adaptation of the questionnaire involved translation of the questionnaire into *Luganda* the language commonly used in central Uganda, where Mulago hospital is located. It was then back-translated into English. Both the *Luganda* and English versions were pre-tested on a sample of 35 mothers to check for applicability and understanding of the questionnaire items and time taken to administer the questionnaire. This process also helped to identify items that needed to be added onto the questionnaire to suit the local setting. The necessary changes were made and the final Ugandan APQ questionnaire consisting of 88 items was developed (Additional File 1).

### Diagnosis of asthma

The diagnoses were based on a systematic approach of the history, clinical examination, laboratory results and chest radiographs, as well as response to short-acting bronchodilators. The details of the laboratory methods have been described previously^39^. Briefly, total white cell and differential counts, and C-reactive protein (CRP) were done to screen for possible viral or bacterial pneumonia. Blood culture was done for diagnosis of bacterial pneumonia and a nasopharyngeal swab for isolation of RSV using immunofluorescence technique. A chest X-ray was done to try and distinguish pneumonia from asthma. An expert panel of three senior pediatricians with experience in pediatric pulmonology and infectious diseases reviewed the case record form for each participant. The experts had no access to the participants. Hence, the diagnoses were made post hoc. Each expert studied the case record form of the participant and, guided by the study definitions, made a diagnosis of asthma or otherwise (pneumonia, bronchiolitis), which was then discussed by all the three experts. A final diagnosis was made if there was concordance between at least two of the three experts. Where there was discordance between all the three experts, the case record forms were subjected to further discussion until a diagnosis was agreed upon. All discussions and conclusions were guided by international case definitions of asthma in children^37^. The participants were assigned one of the following diagnoses based on the criteria; asthma, bronchiolitis, bacterial pneumonia, combined asthma and bacterial pneumonia, viral pneumonia and other (pneumocystis jirovecii pneumonia, pulmonary tuberculosis). The details of the diagnostic criteria (Additional File 2) have been published^1^. RN took the minutes during these proceedings but did not participate in the discussions.

Prior to conducting the study, we performed a literature review and consulted experienced clinicians to generate a study definition of asthma^37,41–43^. This definition was based on a combination of the clinical history, examination findings, results of tests and response to treatment. Briefly, the definition of asthma was based on the Global Initiative for Asthma (GINA) guidelines^37^ that was modified as follows: in the history we excluded “recurrent chest tightness” as a symptom because it is not easily expressed by U-5s^44,45^. We also excluded peak expiratory flow measurements and spirometry because U-5s are not able to perform these tests effectively^46^. Furthermore, we included chest X-rays to help us distinguish asthma from pneumonia. Bronchiolitis was defined as a first episode of wheezing with cough and/or difficult breathing in association with respiratory distress^47^.

The term “Asthma syndrome” was used to refer to children with asthma and bronchiolitis because, among young children, it is difficult to differentiate asthma from bronchiolitis due to the overlap in clinical presentation and uncertainty in recordings of the medical history^2^. Using the label ‘asthma’ essentially implies a similar approach to treatment for children with asthma and bronchiolitis. In addition, asthma is a chronic illness, often starting in infancy^48,49^ and characterized by continuous review, hence, the use of the term asthma syndrome affords an opportunity for re-evaluation of the children with asthma symptoms and eventual identification of those with asthma.

### Statistical considerations

For each of the items in the questionnaire, the sensitivity, specificity, area under the curve (AUC), area under the receiver operating characteristic (ROC) curve (the mean of the sensitivity and specificity), Positive Predictive Value (PPV), Negative Predictive Value (NPV) and misclassification error (MCE) defined as the proportion of children wrongly classified with regard to asthma in the present population, were calculated with 95% CI. We sought to improve the performance of the single items by devising a diagnosis based on a combination of items. A diagnosis based on a combination of items is readily defined from the data at hand as for each possible combination of answers to return the diagnosis corresponding to the most prevalent gold standard diagnosis. In order to investigate the performance of a diagnosis approach based on a combination of items, we investigated all combinations of four items. The combinations with the best performance give an upper limit to the performance of such approach based on combinations of items.

To assess an item with both sensitivity and specificity of at least 80%, in a population with an estimated asthma prevalence of 46%, a sample of 534 children was required^3,50^. Data were double-entered in Epidata version 3.0; analyses were done in SAS version 9.4 and R version.

### Ethical considerations

Ethical approval was obtained from the Higher Degrees, Ethics and Research Committee (HDREC) at Makerere College of Health Sciences and the Uganda National Council of Science and Technology (UNCST). Informed written consent was obtained from the caretakers of the children.

### Reporting summary

Further information on research design is available in the Nature Research Reporting Summary linked to this article.

## Supplementary information

Supplementary Information

Reporting Summary

## Data Availability

The dataset that supports the results in this study is available from the corresponding author on reasonable request.
