# Supplementary material for: Symptom-based screening tool for asthma syndrome among young children in Uganda
Source: NPJ Prim Care Respir Med. 2020 May 6;30:18. doi: 10.1038/s41533-020-0175-1 (PMC7203121; doi:10.1038/s41533-020-0175-1)
Supplement: Supplementary file 1 — Supplementary Information [file 41533_2020_175_MOESM1_ESM.pdf]

Asthma –Pneumonia Questionnaire -APQ (Additional file 1)

Patient Identification

Date		
Study number/code		
Date of birth /age(in months)		
Sex	M	F
Address	Village/cell/LC I	
	Parish/LCII	
	Sub-county/Division/LCIII	
	District	
	Telephone contact	
	Urban	Rural

**A**	**Your child's disease. How did it begin:**	Yes	No	Don't Know
q2	Did the disease start gradually over some days?			
q3	Did the disease start with cold or nose symptoms?			
q4a	Did the disease specifically start with much cough? OR			
q4b	Did the cough come late in the course of disease?			
q4c	Did the cough gradually worsen over some days?			
q5	Did the disease specifically start with changes in breathing? If yes,			
	q5a) Difficulty in breathing			
	q5b) Fast/rapid breathing			
	q5c) Rattling/gurgling			
	q5d) Wheezing/whistling			
q6	Did he/she get sudden high fever?			
q7	Did he/she suddenly become very ill/much worse?			
q8	If your child got pain killers, did it help much?			

**B**	**Your child's disease today:**	Yes	No	Don't Know
9	Does your child have;			
q9a	Cough?			
q9b	Fast/rapid breathing?			
q9c	Difficulty in breathing?			
q9d	Wheezing/whistling?			
q9e	Rattling/gurgling?			
q10	Fever?			
q11	Vomiting?			
q12	Is your child feeding poorly/unable to feed?			
q13	Is your child generally weak?			

C	**What symptoms has your child had in the last few days (before coming to hospital)?**	Yes	No	Don’t Know
q14a	Significant cough? If yes			
q14b	Was the cough dry? **OR**			
q14c	Was the cough productive?			
q14d	Does your child cough up sputum?			
q15a	Is the coughing worse during the day? **OR**			
q15b	Is the coughing worse at night?			
q16	Did your child wake up because of;<br>a) Coughing?			
q16	b) Difficulty breathing?			
q16	c) Fast/rapid breathing?			
q16	d) Gurgling/rattling?			
q16e	Wheezing/whistling?			
q16f	Shortness of breath?			
q17	Was the child vomiting?			
q18	Was the child able to drink or breastfeed?			
q19	Was the child in pain? If yes,			
q19b	Pain when coughing?			
q19c	Abdominal pain?			
q19d	Headache?			
q19e	Chest pain?			
q20	Was your child tired/lethargic **OR**			
q21	Was your child cranky/fussy?			
q22	Did your child want to lie down and sleep? **OR**			
q23	Tried to be active?			
q24	Did your child have very high fever?			
q25	Did your child sleep very badly?			
q26	Has your child been crying a lot?			

E	**In the last 3 months before coming to hospital, has your child:**	Yes	No	Don’t Know
q28	Had many colds?			
q29	Been coughing most of the time?			
q30	Suffered from several week-long bouts of coughing?			
q31	Coughed very little or not at all?			
q32	Coughed a lot in the night/early morning?			
q33	Woken up from sleep because of coughing?			
q34	Had difficulty in breathing?			
q35	Fast/rapid breathing?			
q36	Wheezing/whistling?			
q37	Rattling/gurgling?			
q38	Been very tired?			
q39	Lost weight?			

F	**Earlier problems (since birth):**	Yes	No	Don't Know
q46	Has your child had recurrent episodes of:<br>a) Cough			
q46	b) Difficulty in breathing			
q46	c) Wheezing/whistling?			
q47	Does your child get many colds?			
q48a	Does your child usually cough much when having a cold?<br>If yes,			
q48b	Is the cough mostly dry? **OR**			
q48c	Is there much phlegm in the airways or/and the child brings up much phlegm?			
q49	Does the cough usually occur in the night/early morning?			
q50	Does the difficulty in breathing usually occur in the night/early morning?			
q51	Does the wheezing usually occur in the night/early morning?			
q52	Does your child wake up because of cough?			
q53	Does your child wake up because of difficulty in breathing?			
q54	Does your child wake up because of wheezing?			
qadded	Has your child been to a clinic/health centre/hospital because of much cough? If yes,<br>How many times?			
q57a	Does your child have/ever had any allergies? If yes,			
q57b	Allergic rhinitis?			
q57c	Eczema?			
q57d	Allergic conjunctivitis?			
q58a	Are there any family members who have/have ever had asthma? If yes,			
q58b	Mother			
q58c	Father			
q58d	Siblings			
q60	Has your child ever suffered from ear infections?			
q61	Has your child ever taken medicine for asthma or wheezing?			
q68g	Does any family member has/ever had allergy			

Study definitions for asthma, bronchiolitis and pneumonia (Additional file 2)

Diagnosis	Criteria
**Asthma**<br>High probability if 4 of 5 are present	Cough, wheeze, difficulty in breathing<br>***(at least one)***
	i) Recurrent cough, wheeze and/or difficulty in breathing, ii) positive history of atopy in child (eczema, rhinitis, food, conjunctivitis), iii) history of asthma in first degree relative ***(at least one)***
	Fast breathing, chest indrawing, prolonged expiration, rhonchi ***(at least 3)***
	Good response to bronchodilators
	Chest x-ray: normal or hyperinflation
**Bronchiolitis**<br>Highly probable if 1 and any other criteria are present	Age less than 2 years, cough, difficulty in breathing, index episode of wheeze<br>(***all must be present***)
	Fast breathing, prolonged expiration, chest indrawing, rhonchi ***(at least two)***
	Total white cell count $\leq 15 \times 10^9$cells/l,<br>CRP < 40mg/l, positive RSV ***(at least one)***
	Chest x-ray: normal or hyperinflation
**Bacterial pneumonia**<br>Highly probable if 4 of 5 are present	Fever, cough, difficulty in breathing ***(at least two)***
	Axillary temperature ≥38°C, fast breathing, chest indrawing ***(at least 2)***
	CRP ≥ 40mg/l, total white cell count $\geq 15 \times 10^9$ cells/l, Neutrophils ≥65%<br>***(at least one)***
	Positive blood culture
	Chest x-ray: alveolar infiltrates, consolidation, pleural effusion<br>(***at least one)***
**Viral pneumonia**<br>Highly probable if 3 of 4 are present	Fever, cough, difficulty in breathing<br>***(at least one)***
	Axillary temperature ≥38°C, fast breathing, chest indrawing ***(at least 2)***
	CRP <40mg/l, total white cell count $< 15 \times 10^9$ cells/l, lymphocytes ≥45%, positive RSV<br>***(at least one)***
	Chest x-ray: normal or diffuse infiltrates

Children who had a combination of clinical and laboratory findings from the categories of bacterial pneumonia and asthma were classified as having combined asthma and bacterial pneumonia.

Table 1: Demographic characteristics of the study participants (N=614)

Diagnosis	Total (n)	Percentage (%)	Age group (months)		Sex		Residence	
			< 12	≥12	M	F	Urban	Rural
Bronchiolitis	125	20.4	109	16	85	40	99	26
Asthma	78	12.7	20	58	42	36	64	14
Combined asthma and bacterial pneumonia	50	8.1	17	33	29	21	37	13
Bacterial pneumonia	167	27.2	77	90	95	72	129	38
Viral pneumonia	163	26.5	92	71	82	81	122	41
*Others	31	5.0	18	13	14	17	17	14
Total	614	100	333	264	347	267	468	146

Pulmonary tuberculosis, Pneumocystis Jirovecii Pneumonia

Table 2: Diagnostic properties of the top-10 (according to AUC) individual APQ items for the diagnosis of asthma syndrome in U-5s

	Sensitivity	Specificity	AUC	PPV	NPV	MCE
	% (95%CI)	% (95%CI)	% (95%CI)	% (95%CI)	% (95%CI)	% (95%CI)
Your child's disease today: wheezing/whistling?	80.2 (75.0-84.8)	84.2 (80.2-87.7)	82.2 (79.1-85.3)	78.1 (72.8-82.8)	85.9 (82.0-89.2)	17.4 (14.6-20.6)
What symptoms has your child had in the last few days (before coming to the hospital): wheezing/whistling?	50.6 (44.4-56.7)	91.1 (87.9-93.8)	70.9 (67.5-74.3)	80.0 (73.4-85.7)	72.5 (68.2-76.4)	25.6 (22.2-29.1)
Your child's disease. How did it begin: wheezing/whistling?	36.8 (31.0-42.8)	94.2 (91.5-96.3)	65.5 (62.3-68.7)	81.6 (73.8-73.8)	68.0 (63.8-72.0)	29.5 (26.0-33.2)
In the last 3 months before coming to hospital, has your child: wheezing/whistling?	34.4 (28.7-40.4)	92.8 (89.9-95.2)	63.6 (60.4-66.8)	77.0 (68.7-84.1)	66.9 (62.7-70.9)	31.3 (27.7-35.0)
Earlier problems (since birth): does your child wake up because of wheezing?	29.6 (24.2-35.5)	95.6 (93.1-97.4)	62.6 (59.6-65.6)	82.4 (73.8-89.3)	66.0 (61.8-69.9)	31.6 (28.0-35.3)
Earlier problems (since birth): has your child had recurrent episodes of wheezing/whistling?	32.4 (26.8-38.3)	91.1 (87.9-93.8)	61.8 (58.5-65.0)	71.9 (63.3-79.6)	65.8 (61.6-69.9)	33.1 (29.4-36.8)
Earlier problems (since birth): does the wheezing usually occur in the night/early morning?	26.5 (21.3-32.1)	95.3 (92.8-97.2)	60.9 (58.0-63.8)	79.8 (70.3-87.4)	64.9 (60.8-68.9)	33.1 (29.4-36.8)
What symptoms has your child had in the last few days (before coming to the hospital): did your child wake up because of difficulty breathing?	60.9 (54.8-66.8)	60.7 (55.6-65.6)	60.8 (56.9-64.7)	52.0 (46.3-57.7)	68.9 (63.6-73.8)	39.3 (35.4-43.2)
Earlier problems (since birth): has your child ever taken medicine for asthma or wheezing?	27.3 (22.0-33.0)	94.2 (91.5-96.3)	60.7 (57.7-63.7)	76.7 (67.3-84.6)	64.9 (60.7-68.9)	33.4 (29.7-37.2)
Your child's disease How did it begin: did the disease specifically start with changes in breathing?	47.4 (41.3-53.6)	73.7 (69.0-78.0)	60.6 (56.7-64.4)	55.8 (49.1-62.4)	66.7 (61.9-71.2)	37.1 (33.4-41.0)

Table 3: The best performing set of four items for detection of asthma syndrome in U-5s with acute respiratory symptoms

Disease started with difficulty in breathing?	Did painkillers help much?	Your child's disease today: wheezing whistling?	Did your child have significant cough in the last few days (just before coming to the hospital)?	n	Indicated diagnosis	Without asthma in data (%)	With asthma in data (%)
No	No	No	No	1	Asthma	0.0	100.0
Yes	No	No	No	2	No Asthma	50.0	50.0
No	Yes	No	No	0			
Yes	Yes	No	No	3	No Asthma	100.0	0.0
No	No	Yes	No	0			
Yes	No	Yes	No	2	Asthma	0.0	100.0
No	Yes	Yes	No	0			
Yes	Yes	Yes	No	0			
No	No	No	Yes	127	No Asthma	86.6	13.4
Yes	No	No	Yes	40	No Asthma	85.0	15.0
No	Yes	No	Yes	147	No Asthma	87.8	12.2
Yes	Yes	No	Yes	34	No Asthma	79.4	20.6
No	No	Yes	Yes	78	Asthma	17.9	82.1
Yes	No	Yes	Yes	76	Asthma	17.1	82.9
No	Yes	Yes	Yes	57	Asthma	31.6	68.4
Yes	Yes	Yes	Yes	47	Asthma	25.5	74.5
